# Clinical Characteristics and Diagnostic Pathways of Emphysematous Cystitis in a University Hospital: A Retrospective Observational Study

**DOI:** 10.1155/aiu/9078899

**Published:** 2026-06-28

**Authors:** Tenei Ohno, Katsumi Shigemura, Kazuki Yanagida, Satoshi Kobayashi, Hazuki Inoue, Masahiro Goto, Kazuki Takei, Hideyuki Minagawa, Itsuki Yoshimura, Tomoyuki Kaneko, Yasufumi Miyake, Hiroshi Oba, Takeo Fukagawa, Ken Kozuma, Tohru Nakagawa

**Affiliations:** ^1^ Department of Urology, Teikyo University School of Medicine, Tokyo, Japan, teikyo-u.ac.jp; ^2^ Department of Urology, Kurashiki Medical Center, Kurashiki, Japan, kchnet.or.jp; ^3^ Department of Emergency Medicine, Teikyo University School of Medicine, Tokyo, Japan, teikyo-u.ac.jp; ^4^ Department of Radiology, Teikyo University School of Medicine, Tokyo, Japan, teikyo-u.ac.jp; ^5^ Department of Surgery, Teikyo University School of Medicine, Tokyo, Japan, teikyo-u.ac.jp; ^6^ Department of Internal Medicine, Teikyo University School of Medicine, Tokyo, Japan, teikyo-u.ac.jp

**Keywords:** emphysematous cystitis, *Escherichia coli*, *Klebsiella pneumoniae*, teaching hospital

## Abstract

**Background and Aims:**

Emphysematous cystitis (EC) is an uncommon but clinically important form of complicated urinary tract infection characterized by gas within the bladder wall and/or bladder lumen. Because EC may present with urinary symptoms, nonspecific systemic symptoms, or incidental computed tomography (CT) findings, its diagnostic process in tertiary hospitals remains clinically relevant. This study aimed to describe the clinical characteristics, diagnostic pathways, microbiological findings, treatment, and outcomes of EC in a university hospital.

**Methods:**

We retrospectively reviewed 45 patients diagnosed with EC based on abdominal CT findings at Teikyo University Hospital between October 2010 and March 2024. Data on clinical presentation, comorbidities, microbiological findings, treatment, and clinical outcomes were extracted from electronic medical records.

**Results:**

A total of 45 patients were analyzed, including 25 males and 20 females, with a median age of 79 years (range: 31–97 years). Diabetes mellitus was present in 21 patients. The most frequently detected urinary pathogens were *Escherichia coli* (*n* = 21), including extended‐spectrum β‐lactamase‐producing strains (*n* = 7), and *Klebsiella pneumoniae* (*n* = 9). In blood culture, *K. pneumoniae* (*n* = 7), *E. coli* (*n* = 5), including extended‐spectrum β‐lactamase‐producing strains (*n* = 2), and *Staphylococcus epidermidis* (*n* = 2) were detected. Most patients were managed conservatively; urinary catheter placement was performed in 35 patients. Regarding EC treatment outcomes, 34 patients recovered or improved, 10 died during the clinical course, and 1 had an unknown or unclear outcome. No deaths were judged to be directly attributable to EC itself. Retrospective review of the clinical course leading to CT detection of EC suggested four diagnostic pathways.

**Conclusion:**

EC occurred mainly in elderly patients with comorbidities and was frequently detected by CT in diverse clinical contexts. These diagnostic pathways are a descriptive summary of how EC was detected in this cohort, rather than a validated prognostic or treatment‐guiding classification.

## 1. Introduction

Emphysematous cystitis (EC) is a rare type of complicated urinary tract infection (UTI), characterized by the presence of gas within the bladder wall and lumen [1–3]. The first documented case of air within the urinary tract dates back to 1671, involving a man with pneumaturia. Infectious emphysema of the bladder was initially reported at autopsy by Eisenlohr in the late 1800s and was later defined as “cystitis emphysematosa” by Bailey in 19,61 {1}. EC is generally associated with patients with diabetes mellitus or chronic steroid use, suggesting that uncontrolled serum or urinary glucose levels may have an aggravating effect on its development [1, 2]. Several risk factors have been associated with EC and severe clinical courses [2, 4]. Diabetes mellitus, chronic kidney disease, neurogenic bladder, urinary tract obstruction, immunosuppression, and advanced age have been reported as important predisposing factors. A recent multicenter retrospective study reported that diabetes mellitus, chronic kidney disease, and neurogenic bladder were significant risk factors for EC and emphasized the need for careful observation because of the tendency toward sepsis and mortality [4].

Recent observational data have also explored scoring systems for mortality risk assessment in patients with EC [5].

From a urological perspective, unmanaged urinary conditions—for instance, large residual urine volume, hydronephrosis, or chronic bladder inflammation due to benign prostatic hyperplasia or neurogenic bladder—in addition to inappropriate antibiotic use, may further predispose patients to EC and antimicrobial resistance (AMR). Management of EC generally consists of prompt antimicrobial therapy targeting common urinary pathogens, urinary drainage when indicated, glycemic control in patients with diabetes mellitus, and treatment of underlying or concomitant infections [1, 3]. Most patients can be managed conservatively, although surgical intervention may be required in complicated or refractory cases, such as bladder necrosis, perforation, or uncontrolled infection [1, 3, 6]. Computed tomography (CT) is considered the most useful imaging modality for detecting gas within the bladder wall and/or bladder lumen, assessing disease extent, and excluding alternative causes of urinary gas such as recent urinary tract instrumentation, trauma, or enterovesical fistula [1, 3, 7]. Therefore, we retrospectively reviewed 45 patients with EC diagnosed at Teikyo University Hospital, a tertiary teaching hospital. This study aimed to describe the clinical characteristics, diagnostic pathways, microbiological findings, treatment, and EC treatment outcomes in this cohort.

## 2. Methods

### 2.1. Study Design

This was a single‐center retrospective observational study conducted at Teikyo University Hospital, a 1078‐bed tertiary teaching hospital in Tokyo, Japan. The study included patients diagnosed with EC between October 2010 and March 2024. Teikyo University Hospital has 471 physicians, 30 departments, and 920 nurses. As a tertiary teaching hospital, the department of urology manages a broad range of urological diseases. In general, malignant diseases represent the majority of the departmental case mix, whereas infectious diseases account for approximately one‐third of the major urological disease burden.

### 2.2. Patients

We retrospectively identified patients diagnosed with EC based on abdominal CT findings at Teikyo University Hospital between October 2010 and March 2024. EC was defined as the presence of gas within the bladder wall and/or bladder lumen on abdominal CT, accompanied by clinical or laboratory findings suggestive of UTI, such as fever, urinary symptoms, pyuria, or positive urine culture. Cases in which intravesical gas was explained by recent urinary tract instrumentation, simple postcatheterization air, trauma, or an obvious enterovesical fistula were not classified as EC.

UTI was clinically diagnosed based on a combination of symptoms suggestive of UTI, urinalysis findings including pyuria when available, urine culture results, systemic inflammatory findings, and the treating physician’s clinical judgment. Because this retrospective study reflected routine clinical practice, UTI was not defined by a single hospital‐wide quantitative urine culture threshold.

EC cases were included in the study database after confirmation based on CT findings, radiology reports, and clinical review by urologists. Patients with missing essential clinical data, including demographic information, laboratory data, microbiological results, treatment information, or clinical outcomes, were excluded.

### 2.3. Data Collection

We extracted the following data from electronic medical records: age, sex, body mass index, presence of diabetes mellitus, comorbidities, clinical presentation leading to diagnosis, laboratory data at diagnosis, urine and blood culture results, antimicrobial therapy, urinary catheter placement, and clinical outcomes.

### 2.4. Microbiological Methods

Blood samples were inoculated into both aerobic and anaerobic blood culture bottles and subsequently incubated in the BD BACTEC FX blood culture system (Becton, Dickinson and Company, Sparks, MD, USA). Bacterial identification was performed by matrix‐assisted laser desorption/ionization time‐of‐flight mass spectrometry (MALDI‐TOF MS) using the VITEK MS system (bioMérieux, Marcy‐l’Etoile, France) for blood cultures and the MALDI Biotyper sirius system (Bruker Daltonics GmbH & Co. KG, Bremen, Germany) for urine cultures. Antimicrobial susceptibility testing was performed using the MicroScan WalkAway 96 plus system (Beckman Coulter, Brea, CA, USA). Minimum inhibitory concentration (MIC) values were interpreted according to the Clinical and Laboratory Standards Institute (CLSI) guidelines (M100, 30th Edition, 2020).

### 2.5. Diagnostic Pathways

In addition to summarizing individual symptoms and laboratory findings at diagnosis, we retrospectively reviewed the clinical course that led to CT detection of EC in each patient. Based on this review, cases were descriptively grouped into four diagnostic pathways: CT performed for nonspecific systemic presentations, CT performed during evaluation of severe underlying diseases or advanced comorbid conditions, CT performed for UTI‐related presentations, and incidental detection on CT performed for unrelated conditions. Nonspecific systemic presentations were defined as presentations without lower urinary tract symptoms or gross hematuria, but with systemic symptoms such as fever, chills, general fatigue, appetite loss, nausea or vomiting, or acute disturbance of consciousness. In these cases, abdominal CT was performed to evaluate the systemic symptoms, and EC was detected on CT.

These pathways were derived from the clinical course leading to diagnosis and were not intended to correspond directly to the symptom categories listed in Table 1. They should not be interpreted as a validated diagnostic, prognostic, or treatment‐guiding classification.

**TABLE 1:** Diagnostic process and laboratory data at diagnosis.

**Diagnostic process**	**Number of cases (%)**

Pain on micturition	3 (7%)
Gross hematuria	6 (13%)
General fatigue	17 (38%)
Fever	10 (22%)
Consciousness disorder	9 (20%)
Incidental	5 (11%)

**Laboratory data at diagnosis**	**Median (range)**

WBC	9800 (900–142,100) μl
CRP	6.8 (0.04–39.22) mg/dL
Cre	1.71 (0.19–11.09) mg/dL
eGFR	31.79 (2.87–269.82) mL/min/1.73m^2^
HbA1c	6.05 (4.3–12.4)%

### 2.6. Clinical Outcomes

Clinical outcomes were evaluated as EC treatment outcomes based on medical records. Outcomes were categorized as recovered/improved, death during the clinical course, or unknown/unclear. Deaths during the clinical course were further reviewed to determine whether they were directly attributable to EC itself or occurred in the setting of underlying or concurrent conditions.

### 2.7. Statistical Analysis

Continuous variables are presented as medians with ranges, and categorical variables are presented as numbers and percentages. Because this was a descriptive retrospective observational study with a limited sample size, no formal hypothesis testing was performed. Accordingly, no *p* values are presented, no a priori level of statistical significance was set, and no 1‐sided or 2‐sided statistical tests were conducted. All analyses were exploratory and descriptive.

### 2.8. Ethical Considerations

The study protocol was approved by the Ethics Committee of Teikyo University School of Medicine (TeiRin 24‐071). The requirement for informed consent was waived by the ethics committee because of the retrospective nature of the study and the use of existing clinical data.

## 3. Results

### 3.1. Clinicopathological Characteristics

A total of 45 cases were analyzed (25 males and 20 females) with a median age of 79 years (range: 31–97 years). Diabetes mellitus was present in 21 patients. Other comorbidities included cerebral infarction (*n* = 6, 13%) and metastatic cancer (*n* = 5, 11%) (Table 2).

**TABLE 2:** Clinicopathological characteristics.

**Characteristics**	**Number of cases (%) or median (range)**

Total	*n* = 45
Age	79 (31–97)
Gender, male	25 (56%)
Female	20 (44%)
BMI (*n* = 36)	20.3 (14.6–33.6)

**Comorbidities/past medical history**	

Diabetes mellitus	21 (47%)
Hypertension	10 (22%)
Chronic kidney disease, G3‐5	5 (11%)
Cerebral infarction	6 (13%)
Coronary heart disease	5 (11%)
Cancer	17 (38%)
Metastatic cancer	5 (11%)

### 3.2. Diagnostic Process and Laboratory Data

The diagnostic process included cases with symptoms reminiscent of UTI, such as pain on micturition (*n* = 3, 7%), gross hematuria (*n* = 6, 13%), and fever (*n* = 10, 22%). There were also symptoms such as consciousness disorder (*n* = 9, 20%) and general fatigue (*n* = 17, 38%). In addition, there were cases where no symptoms were present, but incidental findings were made during CT scans performed for the evaluation of other conditions (Table 1).

### 3.3. Bacteria Detected on Urine Culture

The bacterial etiology in urine culture primarily involved *Escherichia coli* (21 cases, 47%), including 7 ESBL‐producing *E. coli* isolates (16%), and *Klebsiella pneumoniae* (9 cases, 20%), with other organisms such as *Corynebacterium* species (2 cases, 4%), *Lactobacillus* species (2 cases, 4%), *Trichosporon asahii* (1 case, 2%), and *Enterobacter aerogenes* (1 case, 2%). The urine culture results are summarized in Table 3. In blood culture, *K. pneumoniae* (7 cases, 16%), *E. coli* (5 cases, 11%), including 2 ESBL‐producing *E. coli* isolates, and *Staphylococcus epidermidis* (2 cases, 4%) were detected. Some patients had polymicrobial urine cultures; therefore, the total percentage of detected pathogens exceeded 100%. Among patients with positive blood cultures and positive urine culture results, the same organism was detected in both urine and blood cultures in 10 of 14 patients (71.4%). Different organisms were detected in 4 patients (28.6%), and 2 additional patients had positive blood cultures with negative or unavailable urine culture results.

**TABLE 3:** Bacteria detected on urine culture.

**Organism**		**%**

*E. coli*	21	47
(*E. coli* (ESBL))	7	16
*K. pneumoniae*	9	20
*Corynebacterium* species	2	4
*Lactobacillus* species	2	4
*T. asahii*	1	2
*Enterobacter aerogenes*	1	2
Yeast‐like cell	1	2
No growth	3	7
Not performed	7	16

### 3.4. Treatments and Outcomes

The most commonly used first‐choice antibiotic was MEPM (*n* = 11, 24%), followed by CTRX (*n* = 7, 16%), and then PIPC/TAZ (*n* = 3, 9%). There was a tendency to choose broad‐spectrum antibiotics that cover gram‐negative bacilli (Table 4). Most patients were managed conservatively with antimicrobial therapy and/or urinary catheter placement; urinary catheter placement was performed in 35 patients. Regarding EC treatment outcomes, 34 patients recovered or improved, 10 died during the clinical course, and 1 had an unknown or unclear outcome. No deaths were judged to be directly attributable to EC itself based on retrospective review of the clinical records (Table 4). No additional EC‐directed treatments other than antimicrobial therapy and urinary catheterization, such as bladder debridement or cystectomy, were performed in this cohort.

**TABLE 4:** Treatments and outcomes.

**Treatment**	

Urinary catheter placement	35 (78%)
Antibiotics	
MEPM	11 (24%)
ABPC	1 (3%)
CTRX	7 (16%)
CFPM	2 (6%)
LVFX	3 (9%)
CEZ	1 (3%)
PIPC/TAZ	3 (9%)
DRPM + VCM	1 (3%)
None	8 (18%)

**Clinical outcomes as EC treatment outcomes**	

Recovered/improved	34 (75.6%)
Death during the clinical course	10 (22.2%)
Unknown/unclear	1 (2.2%)
Attribution of death among patients who died	
Death directly attributable to EC itself	0/10 (0%)

## 4. Discussion

The cohort mainly comprised elderly patients with multiple comorbidities. This demographic profile is consistent with known risk factors for EC [2, 4], as elderly patients and those with comorbidities, particularly diabetes mellitus, are susceptible to complicated UTIs.

The four diagnostic pathways described in this study should be interpreted as a descriptive summary of how EC was detected in our 45 patients, rather than as a validated classification system. These pathways should also be distinguished from the symptom‐based summary shown in Table 1. Table 1 summarizes individual presenting symptoms and laboratory data at diagnosis, whereas the diagnostic pathways describe the broader clinical context in which CT was performed and EC was detected. Therefore, these pathways do not imply mutually exclusive symptom categories, nor do they demonstrate superiority over existing diagnostic approaches or prove an effect on management decisions or outcomes. Nevertheless, they may be clinically useful by emphasizing that EC can be detected in diverse clinical contexts, including nonspecific systemic presentations, severe underlying diseases or advanced comorbid conditions, UTI‐related presentations, and incidental CT findings. In clinical practice, these pathways may encourage clinicians to consider EC when CT shows bladder wall or intraluminal gas, even when typical urinary symptoms are absent. Accordingly, the clinical usefulness of these pathways should be regarded as hypothesis‐generating and requires validation in future prospective studies.

CT plays a central role in EC diagnosis because it can detect gas within the bladder wall and/or bladder lumen, assess disease extent, and help exclude alternative causes of urinary gas, such as recent urinary tract instrumentation, trauma, or enterovesical fistula [1, 3, 7]. However, CT findings should be interpreted together with clinical and laboratory evidence of UTI, because intravesical gas alone may be caused by noninfectious or non‐EC conditions.

Diabetes mellitus was common in our cohort. Hyperglycemia may promote bacterial growth and impair host immune responses, thereby increasing the risk of gas‐forming infections [1, 2]. The pathogenesis of EC has been linked to gas production by bacteria within the bladder wall and/or lumen.

The microbiological findings in this cohort were dominated by *E. coli* (21 cases, 47%) and *K. pneumoniae* (9 cases, 20%), consistent with typical pathogen profiles reported in EC and complicated UTIs [1, 2, 6]. These organisms can contribute to gas formation through fermentation processes, supporting their pathogenic role in EC [1, 2].

Most cases were managed with antimicrobial therapy and/or urinary catheterization. Antimicrobial therapy for EC should be selected according to disease severity, likely urinary pathogens, local AMR patterns, and culture and susceptibility results when available [1, 3]. In our cohort, broad‐spectrum agents covering gram‐negative bacilli were frequently selected, reflecting the severity and complexity of patients treated in a tertiary hospital.

Previous studies have reported that EC can be associated with sepsis and mortality, particularly in patients with diabetes mellitus, chronic kidney disease, or neurogenic bladder [4, 5]. Choi et al. [4] identified diabetes mellitus, chronic kidney disease, and neurogenic bladder as significant risk factors for EC, and Chen et al. [5] evaluated mortality risk assessment using clinical scoring systems. In the present cohort, 10 patients died during the clinical course; however, no deaths were judged to be directly attributable to EC itself based on retrospective review of the clinical records. These findings suggest that EC‐specific treatment outcomes and overall mortality during the clinical course should be interpreted separately, particularly in tertiary hospital cohorts with substantial comorbidity. The retrospective design and limited sample size preclude any conclusion regarding prognostic benefit.

Because this study was conducted in a tertiary teaching hospital, the cohort included many elderly patients with malignancy, cardiovascular disease, chronic kidney disease, and other complex comorbidities. This institutional setting may explain why overall deaths during the clinical course occurred despite the absence of deaths judged to be directly attributable to EC itself. Therefore, the generalizability of our findings to community hospitals or less complex patient populations may be limited.

### 4.1. Limitations

This study has several limitations. First, it was a single‐center retrospective study with a limited sample size. Second, disease severity scores, such as those evaluated by Chen et al. [5], were not available for all patients. Third, because this was a descriptive study, detailed statistical analyses of predictors of treatment failure or mortality were not performed. Fourth, although initial and subsequent antimicrobial agents were recorded, detailed and standardized information on the timing and rationale for antimicrobial changes, including whether changes represented de‐escalation based on culture and susceptibility results, was not consistently available. Finally, attribution of death was based on retrospective review of clinical records and may be affected by severe comorbidities and concurrent conditions. These limitations should be addressed in future prospective multicenter studies.

## 5. Conclusion

*E. coli*, including ESBL‐producing strains, and *K. pneumoniae* were the primary etiological agents in this cohort. EC was detected through diverse clinical contexts in a tertiary teaching hospital, and the four diagnostic pathways described in this study should be interpreted as a descriptive summary of how EC was detected rather than as a validated prognostic or treatment‐guiding classification. No deaths were judged to be directly attributable to EC itself, although deaths occurred during the clinical course in patients with severe underlying or concurrent conditions. Further prospective multicenter studies incorporating disease severity, gas extent, and patient risk factors are warranted to refine risk stratification and management strategies.

## Author Contributions

Drs. Tenei Ohno and Katsumi Shigemura had full access to all of the data in this study and takes complete responsibility for the integrity of the data and the accuracy of the data analysis.

## Funding

This research received no specific grant from any funding agency in the public, commercial, or not‐for‐profit sectors.

## Disclosure

No funding source or financial relationship had any role in the study design; collection, analysis, or interpretation of data; writing of the manuscript; or the decision to submit the manuscript for publication. All authors have read and approved the final version of the manuscript.

## Ethics Statement

The study protocol was approved by the Ethics Committee of Teikyo University School of Medicine (approval number: TeiRin 24‐071).

## Consent

The requirement for informed consent was waived by the ethics committee because of the retrospective nature of the study and the use of existing clinical data.

## Conflicts of Interest

The authors declare no conflicts of interest.

## Data Availability

The data that support the findings of this study are available on request from the 1st and corresponding authors. The data are not publicly available due to privacy or ethical restrictions.
